# Elevated germline mutation rate in teenage fathers

**DOI:** 10.1098/rspb.2014.2898

**Published:** 2015-03-22

**Authors:** Peter Forster, Carsten Hohoff, Bettina Dunkelmann, Marianne Schürenkamp, Heidi Pfeiffer, Franz Neuhuber, Bernd Brinkmann

**Affiliations:** 1 Institute for Forensic Genetics, Münster 48161, Germany; 2 Murray Edwards College, University of Cambridge, Cambridge CB3 0DF, UK; 3 Institute of Legal Medicine, University of Salzburg, Ignaz-Harrer-Strasse 79, Salzburg 5020, Austria; 4 Institute of Legal Medicine, University of Münster, Münster 48149, Germany

**Keywords:** ageing, immortality, stem cell, spermatogenesis, oogenesis, molecular clock

## Abstract

Men age and die, while cells in their germline are programmed to be immortal. To elucidate how germ cells maintain viable DNA despite increasing parental age, we analysed DNA from 24 097 parents and their children, from Europe, the Middle East and Africa. We chose repetitive microsatellite DNA that mutates (unlike point mutations) only as a result of cellular replication, providing us with a natural ‘cell-cycle counter’. We observe, as expected, that the overall mutation rate for fathers is seven times higher than for mothers. Also as expected, mothers have a low and lifelong constant DNA mutation rate. Surprisingly, however, we discover that (i) teenage fathers already set out from a much higher mutation rate than teenage mothers (potentially equivalent to 77–196 male germline cell divisions by puberty); and (ii) ageing men maintain sperm DNA quality similar to that of teenagers, presumably by using fresh batches of stem cells known as ‘A-dark spermatogonia’.

## 1. Introduction

In 1912, Weinberg [1] examined cases of achondroplasia (a form of dwarfism) and observed that last-born children were more likely to be affected than younger siblings. This indicated that the disease was at least partly owing to new mutations in the germline of the parents, and that the mutation rate depended on parental age. Penrose [2] offered the explanation that ‘There are very few cell divisions in the female germ line but many in the male germ line since the spermatogonia are continuously dividing. Thus the incidence of mutation due to failure to copy a gene at cell division would be unlikely to have any strong relation to maternal age; a marked increase of defects with this origin, however, would be seen at late paternal ages’ (p. 312). Vogel & Motulsky [3] estimated that female and male germ cells undergo 22 and 30 divisions, respectively, before puberty. From an average age of fifteen onwards, the male germ cells would initially undergo 23 divisions a year, decreasing with age.

Since then, empirical evidence for and against Penrose's hypothesis has emerged. On the one hand, it is true that the paternal mutation rate is consistently observed to be six to nine times higher than the maternal mutation rate, depending on the loci investigated [4–6]. On the other hand, while the incidence of certain *de novo* dominant genetic diseases does increase with paternal age, the increase becomes progressively steeper [3]. The apparent acceleration is caused by specific mutations that bestow a selective advantage on spermatogonial stem cells that carry these mutations, similar to a cancer [7]. Genes that convey such selective effects are the exception rather than the rule [8]. Moreover, reproductive biologists have moved away from a simple chain of descent of spermatogonia as a model of how spermatogonial stem cells give rise to sperm, and have instead invoked nonlinear progenitor cells (A-dark and A-pale spermatogonia), which will be discussed later.

From a forensic point of view, a little-discussed problem in such classical family studies is the effect of undisclosed non-paternities—older men are less likely to be fertile, increasing the probability of an illegitimate child by the potentially younger wife, and any genetic difference between the father and the illegitimate child might then be misinterpreted as a *de novo* mutation. This point is relevant to historical studies, which did not include paternity testing.

In order to explore the influences of age and sex on the human germline, we have analysed short tandem repeats (STRs, i.e. microsatellites) from 24 097 normal parents and their validated biological children in residents of Germany, Austria, the Middle East and West Africa.

**Figure 1.**
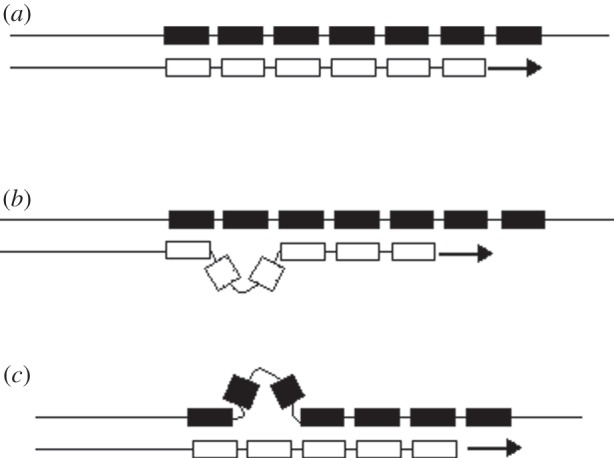
DNA strand slippage during replication of an STR locus. The boxes symbolize repetitive DNA units, typically tetramers such as GATAGATAGATA, which together constitute the STR locus. The arrows indicate the direction in which a new DNA strand (white boxes) is being replicated from the template strand (black boxes). Three different situations during DNA replication are depicted. (*a*) The DNA replication of the STR locus has proceeded without a mutation. (*b*) The DNA replication of the STR locus has led to a gain of one repeat unit owing to a loop in the new strand; the aberrant loop is stabilized by flanking repeat units complementary to the opposite strand. (*c*) The DNA replication of the STR locus has led to a loss of one repeat unit owing to a loop in the template strand; the aberrant loop is likewise stabilized by flanking repeat units.

We chose to analyse STRs [9,10] because (i) they mutate 100 000 times faster than single nucleotides and indeed 1 million times faster in the case of the ACTBP2 locus we include here; (ii) their multiallelic variation allows them to be conveniently traced to the parent of origin; and most importantly, (iii) they mutate only during cellular replication [11], providing us with a mutational ‘cell-cycle counter’ that counts the number of cell divisions since the tissue or human being under investigation originated from an ancestral cell (figure 1).

## 2. Subjects and methods

### (a) Subjects

In the course of routine paternity testing, blood or saliva samples were provided by subjects living in Germany (Münster area) and Austria (Salzburg area) between 1990 and 2010. DNA was extracted either from venous blood samples for those samples in the Münster set that predated March 2000, or from saliva samples dried on cotton buds (all others). Blood samples were taken only from German residents sampled before March 1999, after which the Münster laboratory switched to saliva sampling. In other words, Austrians and immigration cases throughout were sampled only for saliva. Sons (51.3%) slightly outnumbered daughters (48.7%). DNA mutations found in saliva samples, i.e. epithelial cells, largely reflect germline mutations [12] passed down to children and grandchildren, as opposed to somatic mutations restricted to certain tissues, as found in disease studies [13].

### (b) Short tandem repeat profiling

The 32 loci chosen for typing are listed in table S1 in the electronic supplementary material, and consist of up to 29 STR loci and three variable number of tandem repeat (VNTR) loci per person. On average, 12.5 loci were typed per individual. DNA typing of all 301 335 alleles, and additional DNA sequencing of those alleles affected by mutations, were performed as previously published [4,6,14–16].

Briefly, genomic DNA was extracted from oral cotton swab samples by the proteinase K/Chelex method [17]. The STR loci were amplified using various kits: AmpFISTR Profiler, SEFiler and Identifiler (Applied Biosystems, Darmstadt, Germany), Power ES (Promega, Mannheim, Germany) and MPX3-SE (Serac, Bad Homburg, Germany). The resulting polymerase chain reaction products were analysed using denaturing capillary gel electrophoresis on ABI PRISM 310 or 3100-Avant Genetic Analyzers according to the manufacturer's instructions.

To independently confirm (i) unusual alleles (‘variant’ alleles often lacking one or more nucleotides in a repeat unit) and (ii) *de novo* mutations and (iii) whether the mutation had occurred in the repeat array rather than in the flanking DNA, variant alleles and alleles from the mutant families were isolated as described elsewhere [18] and were subjected to direct sequencing using the BigDye Terminator Cycle Sequencing Kit (ABI) with primers for both strands. Thus, in all cases of statistically confirmed mutations, all alleles in the father, mother and child at the locus involved were sequenced. No nucleotide point mutations were found in our approximately 400 mutation cases; this is as expected, given that STR amplicons are on the order of 100–200 nucleotides long, and that their mutation rate is from 100 000 to 1 million times faster than that of single nucleotides. As a desired side effect, this intra-locus sequencing was often sufficient for assigning a new mutation to a parent of origin, specifically in complex STR loci, where the overall allele length might be identical in the two parents, while the detailed sequence revealed from whom the child had inherited the mutant allele.

Among the remaining doubtful cases where the parental origin of the mutation was unclear, we typed flanking STR loci and used these to construct family-specific haplotypes [6]. The selection criteria for these flanking loci were their genetic distance (up to approx. 8 cM) and high polymorphism. Five to seven polymorphic flanking markers upstream as well as downstream were selected for each of the four most highly mutated loci D3S1358, FGA, ACTBP2 and VWA. Each amplicon length was regarded as an allele and used to construct family-specific haplotypes as described by Klintschar *et al.* [19].

### (c) Statistical analysis

Hardy–Weinberg expectations were calculated with the in-house computer programme HWE-Analysis 3.2 (Christoph Puers, Münster), which confirmed that the allele frequency distribution at nearly every locus studied was consistent with Hardy–Weinberg equilibrium conditions. One exception was the TH01 locus on chromosome 11p15. This is a locus linked to type I diabetes, which we discovered was subject to transmission distortion [20].

Familial relationship probabilities were calculated according to Essen-Möller and Quensel [21] and International Society for Forensic Genetics (ISFG) recommendations [22]. Briefly, whenever a mismatch at only one locus, and thus a potential *de novo* mutation, was found between a parent and a child, further STRs were typed. If the mismatch remained isolated, statistical analysis of the probability of the respective parenthood was performed. Taking a conservative stance, the discrepant locus was always included in the calculation [23], hence the overall parenthood probability decreased significantly after this step, by two or more orders of magnitude. The assumption of a new mutation was made only if the final probability, that is after inclusion of all loci typed, attained or exceeded 99.97%.

Linear least-squares regression analysis on maternal and paternal ages versus mutation rates was performed using OriginPro 8.5 (http://www.originlab.com/index.aspx?go=PRODUCTS/OriginPro), applying instrumental weighting to take into account the level of confidence in each data point. The confidence in the data point is inversely proportional to the square of the error value. For the linear regression as a whole the confidence interval of 95% was calculated, which defines the standard errors for intercept and slope.

## 3. Results

### (a) Eliminating the non-paternity rate in humans

In the course of routine paternity testing, blood or saliva samples were collected between 1990 and 2010 from subjects living in Germany and Austria, and were typed for up to 32 repetitive DNA loci. Most of these Germans or Austrians are natives from the Münster and Salzburg areas, respectively. Additionally, in the course of routine immigration testing to facilitate family reunions, saliva samples of predominantly Kurdish individuals from the Middle East and from African individuals predominantly from Nigeria and neighbouring countries were taken in the period 1997–2010 and typed for up to 29 STR loci.

Unlike the European families, who were routine paternity cases, the West African and Middle Eastern families requested voluntary typing at considerable personal expense to enable separated family members to immigrate to Germany. Thus, with the West African and Middle Eastern typing results, we can estimate the percentage of paternally unsuspected illegitimacy: the values range from 1.6% in Middle Easterners to 8.2% in Africans. By comparison, a 1.3% unsuspected non-paternity incidence has been measured in native Englishmen [24]. These frequencies are one to two orders of magnitude higher than a genuine mutation event at an average STR locus, underlining the need for careful paternity testing in any pedigree or epidemiological studies attempting to examine germline mutation effects in humans.

### (b) *De novo* mutations predominantly in the male germline

In 301 335 parent–child allele transfers, we observed 401 mutation events, which are summarized in table 1 and electronic supplementary material, table S1, and we independently confirmed each observed mutation by DNA sequencing.

**Table 1:** Parents' ages, analysed meioses and observed mutations. Ages refer to parental age at conception of child. Mutations in all but six cases refer to single-repeat mutations; the six double mutations are counted as two mutations each. The two entries ‘50/2’ mean there are 50 mutations in total that cannot be assigned to the mother or the father despite sequencing. ‘n.a.’ refers to rare cases where the mutant parents' ages were not available and hence the number of analysed meioses are not applicable for the purpose of age-specific mutation rate calculations.

age class	fathers	mothers	paternal meioses	maternal meioses	certainly paternal mutations	certainly maternal mutations	potentially paternal mutations	potentially maternal mutations
10–14.9y	42	154	532	1946	0	0	0	0
15–19.9y	1070	2653	13 308	32 967	23	14	6	10
20–24.9y	2760	4197	34 585	52 475	52	18	9	15
25–29.9y	3043	2995	38 285	37 442	67	10	13	6
30–34.9y	2305	1684	29 034	20 937	74	9	9	10
35–39.9y	1286	699	16 121	8706	39	2	5	4
40–44.9y	614	146	7742	1841	20	0	5	3
45–49.9y	263	16	3279	203	7	0	1	0
50–54.9y	92	2	1141	25	5	0	0	0
55–59.9y	36	0	437	0	1	0	0	0
60–64.9y	24	0	274	0	1	0	1	0
65–71y	4	0	55	0	0	0	0	0
age n.a.	9	3	n.a.	n.a.	8	1	1	2
totals	11 548	12 549	144 793	156 542	297	54	50/2	50/2

As expected for autosomal loci, we found that sons and daughters were equally likely to be mutated. All of the mutations were single-repeat mutations, except for six double-repeat mutations, as postulated by Weber & Wong [25]. Overall, repeat gains (where the allele was lengthened by one repeat unit) nearly equalled losses; 171 mutations were gains while 172 were losses, with the remainder not assignable. The few early blood samples yielded a slightly higher mutation rate than the predominant saliva samples (among a subset of directly comparable STR loci: 20 mutations in 4938 meioses typed from blood, compared with 19 mutations in 7803 meioses typed from saliva) but the difference is not significant (*p* = 0.137, Fisher's exact test). Fifty-four of the mutations were found to originate from the mothers among 156 542 maternal transfers, 297 mutations from the fathers among 144 793 paternal transfers and 50 mutations could not be assigned, in cases where even DNA sequencing did not provide resolution of the parental origin or where sample material was exhausted, particularly in older casework. For further analyses, each of these unassigned mutations was scored as 0.905 paternal mutations and 0.095 maternal mutations, respectively, according to our quantitative study [6].

### (c) Mutation rate as a function of sex and age

**Figure 2.**
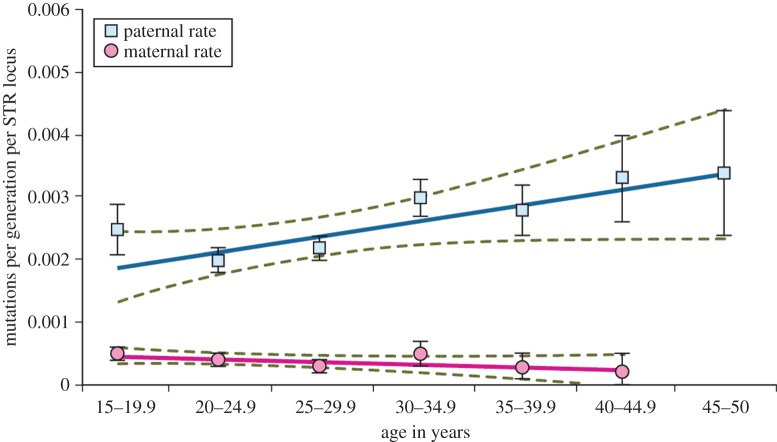
Germline STR mutation rates for fathers (blue) and mothers (pink). Ages refer to the parent's age at conception, starting with 15 years and ending with 45 years for mothers and 50 years for fathers. The straight lines are linear regressions and the accompanying green lines are the 95% confidence intervals (CIs). Standard deviations for individual values are shown as bars. The graph is based on 325 paternal mutations determined from 142 354 meioses, and on 58 maternal mutations determined from 154 368 meioses.

The parental ages used here pertain to conception and were calculated by subtracting 270 days from the day of birth of the child. We identified the youngest mother as being 10.7 years old and the oldest mother as 52.1 years old in our sample of 12 549 mothers. Among the 11 548 biological fathers, the youngest age was 12.1 years and the oldest father was 70.1 years at conception. After grouping the parents into 5-year intervals, starting at 15 years and ending at 45 years (for mothers) and 50 years (for fathers) to avoid insufficient sample sizes, we determined the mutation rate as a function of age, as shown in figure 2.

Looking at the details of figure 2, there are three different mutation rates that will be particularly relevant.

First, in agreement with the concept that females have a store of oocytes that do not require further replication before fertilization, the maternal mutation rate remains constant at a low level, on average 0.37 × 10^−3^ ± 0.05 × 10^−3^ STR mutations per locus per generation.

Second, the fathers superficially also seem to agree with the classic concept that continuous replication in spermatogenesis induces errors: the fathers' mutation rate overall is about six times higher than the mothers' mutation rate. On closer inspection of figure 2, however, the male mutation rate clearly does not commence at the low female level among the teenage parents. This would have been expected if only 22 and 30 cell divisions had occurred in the female and male germline, respectively, by the age of 14 [3] Rather, the male mutation rate appears to start on a five times higher plateau at 1.9 × 10^−3^ STR mutations per locus per generation (95% confidence interval (CI) 1.3–2.4), if we trust the linear regression in teenage parents. More realistically, if we instead accept clinical studies that offspring of teenagers do have a slightly elevated medical risk compared with offspring of parents in their twenties [26–30], our realistic teenage point estimate is 2.5 × 10^−3^ mutations per generation (95% CI 1.6–3.3), resulting in a 6.7-fold (95% CI 3.5–8.9) higher mutation rate for teenage fathers than mothers.

Third, the fathers' mutation rate then rises only slightly by age 50 to 3.4 × 10^−3^ (95% CI 2.4–4.4) STR mutations per locus per generation. Hence, we observe an unexpectedly modest mutation rate increase of only around 1.3-fold between teenage fathers and 50-year-old fathers.

## 4. Discussion

### (a) Confirmation of cell-cycle dependency of short tandem repeat mutations

Unlike point mutations, STR mutations, which we have analysed here, are thought to reflect DNA replication errors [11] rather than external chemical or physical mutagenesis. This assumption is strongly supported by our observation that the low female mutation rate does not measurably increase with age (figure 2).

### (b) Unexpected cell divisions in pre-puberty spermatogenesis?

While a low and constant maternal mutation rate is expected on the basis of Penrose's classic genetic proposal and Vogel and Motulsky's considerations, the shallow increase with paternal age is not, nor is our discovery of a much higher male than female mutation rate even among teenage parents. Specifically, if we assume that Vogel & Motulsky [3] have accurately estimated oogenesis to require 22 cell divisions between the conception of a female embryo and her puberty, and if the replication error rate per cell division is the same between male and female germ cells, then from the teenage fathers' versus mothers' mutation ratio of 6.7-fold (95% CI 3.5–8.9), it follows that approximately 6.7 × 22 = 147 (95% CI 77–196) cell divisions are needed in the complex process of spermatogenesis until puberty. This is considerably more than the 30 divisions estimated by Vogel and Motulsky, which they tentatively extrapolated from histological and volumetric considerations.

### (c) DNA quality in aging fathers' germline

More critically, if Vogel and Motulsky are correct in estimating that male germ cells, from an average age of 20 years onwards, initially undergo 23 divisions a year, then it would follow that the male germline by age 50 would undergo 23 × 30 = 690 cell divisions, and therefore a mutation rate 690/22, that is 31 times higher than the female mutation rate and thus nearly six times higher than the teenage fathers' mutation rate, even assuming a linear rather than an exponential process. In fact, we observe that the mutation rate at paternal age 50 is not six times but only 1.3 (95% CI 0.7–2.7) times higher than for teenage fathers. Considered from an evolutionary perspective, the observation that humans maintain a low mutation rate despite advancing paternal age is perhaps unsurprising, as it enables immortality of the germline in the species.

### (d) Life expectancy of 120 days or eight cycles for sperm progenitor cells

Clearly, therefore, the notion of a constantly replicating pool of progenitor cells during spermatogenesis may have to be discarded in favour of a mechanism involving largely dormant progenitor or stem cells in spermatogenesis. Precisely, such stem cells have in fact been postulated to be ‘A-dark spermatogonia’ [31,32], which form a reserve of stem cells, and when required, produce ‘A-pale spermatogonia’ that normally differentiate into spermatocytes.

Using our observed paternal STR mutation rates as a ‘cell-cycle counter’, and assuming a model where, from puberty onwards, a dormant progenitor stem cell (putatively an A-dark cell) undergoes cell division to produce one dormant stem cell and one active progenitor cell (putatively an A-pale cell) that differentiates, we can estimate the lifespan of the postulated stem cell once it is activated for spermatogenesis: the slope of the regression is 0.000248 with a standard error of 9.3 × 10^–5^ mutations per 5 years in figure 2, hence 0.0000496 with a standard error of 1.9 × 10^–5^ mutations per year. In teenage fathers, we expect one STR mutation to have occurred per 147/0.0025 cell divisions (95% CI 77/0.0033–196/0.0016), based ultimately on the reliably known 22 cell divisions during oogenesis as a calibration benchmark. Our observed number of STR mutations per year during the fathers' reproductive lifetimes thus corresponds to 2.94 (95% CI 0.3–8.3) cell divisions per year (147 divisions × 0.0000496/0.0025), therefore, a stem cell activated for spermatogenesis would have a life expectancy of 124 days (365/2.95 days), with a 95% CI of 44–1135 days. Our result of about 124 days for the life expectancy of the postulated spermatogenic progenitor cell, based on our STR ‘cell-cycle counter’, contrasts with Heller & Clermont's [31] report of 16 days' division cycle for the spermatogonia. We conclude therefore that the postulated stem cell would be dormant, while about eight (124/16, 95% CI 3–71) spermatogonial cycles produce mature spermatocytes.

### (e) Exploring alternative explanations: elevated pre-puberty DNA mutation rate

So far, we have assumed the notion of a clock-like STR mutation rate during male development, which indicates a higher number of cell divisions during spermatogenesis. However, it could be argued that it is not the cell division tally but instead the DNA mutation rate that is elevated it the male zygote. This interpretation might be justified by reference to irradiation experiments on mice, which show that the male and female pronuclei are asymetric regarding damage and repair mechanisms [33]. Most or all of the proteins and mRNAs used in repair at this stage are supplied necessarily by the oocyte. Based on staining of markers for double-strand breaks, the paternal pronucleus appears to have many more double-strand breaks. It would then stand to reason that, to the extent that homology-driven mechanisms are used to repair the double-strand breaks at this developmental stage, the template would be supplied by the maternal genome. We have therefore consulted our data whether indeed the mutant paternal allele in a child typically becomes identical to the allele inherited from the mother. It transpires that this is clearly not the case: for our most informative locus, ACTBP2, there are 59 paternal mutant alleles in the children, and only two of them have become identical to the mother's alleles. Lowering our expectations, we might instead try to salvage this hypothesis by arguing that the maternal allele is not simply copied via homologous recombination, but that the mutant allele at least becomes more similar to the maternal allele in terms of repetitive DNA length. Again, our data show this is not really the case: out of the 59 paternal mutations, 27 mutations make the child more different from the mother, while 32 mutations make the child more similar to the mother in terms of allele length. This is not significantly different from the expected chance value of 59/2. And also among the teenage fathers, the non-homologies to the mother's allele outnumber the homologies by three to one. We conclude that human STR mutations are more likely to reflect the cell division history rather than a hypothetical increased mutation load in the male pronucleus (increased compared with the female pronucleus) that would then be expected to be repaired by homologous recombination with the maternal allele.

However, the lack of a signal still does not rule out male-specific mutation processes. Is it true without exception that double-strand breaks in the male pronucleus are repaired by homology-driven mechanisms using the female genome as a template? Also, could there be ‘one-off’ mutation events during spermatogenesis, such as during meiosis or spermiogenesis, that would not show age-related increases in frequency since these events only happen once during the making of a sperm, regardless of age? We are not aware of positive evidence for such more elaborate scenarios, but they should be borne in mind, as they would modify or invalidate our lifespan calculations for spermatogonial cells.

We suggest that qualitative support for our preferred explanation (that STR mutations act as a cell-cycle counter, whereas point mutations are the sum of both spontaneous and replication-dependent mutation events) comes from two mutation studies in adults, analysing STR mutations [34] and point mutations [35]. The former authors, in their note added in proof, were intrigued to find that ‘In spite of some key similarities between our [STR] results and those [point mutation results] of Kong *et al*. [2012] they estimate a considerably stronger effect of father's age’ ([35], p. 1164). Indeed, between 20- and 40-year-old fathers, we find a 1.3-fold increase in STR mutation rate (similar to Sun's factor of 1.5 [32]), while by contrast Kong *et al.* [35] find a doubling of the single nucleotide mutation rate for the same age range in their fig. 2. Kong *et al*.'s twofold increase is lower than the three- to fourfold increase we would expect in our simplistic application of the Vogel–Motulsky proposals [3], and part of the discrepancy is evidently owing to the fact that their graph plots fathers' ages versus maternal and paternal mutations combined, rather than just versus paternal mutations, thus partly obscuring the paternal increase. Another caveat is that Kong's sample was preselected for schizophrenic and autistic patients. Tentative support for our proposed STR cell-cycle counter may seem to come from the maternal data of Kong *et al.* [35]: while our STR mutations do not increase with maternal age, the point mutations in their mothers seem to double between the ages of 20 and 35. Although this is encouraging, an important caveat here is that their sample consisted of only five mothers. Furthermore, a study modelling mutations in 250 Dutch pedigrees [36] does not find a significant correlation between mother's age and maternal point mutations. However, a recent American conference report [37] on a larger sample does find a slight but significant increase in maternal point mutations, in line with our expectations.

### (f) Outlook for developmental and cancer research

Here, we have applied our STR ‘cell-cycle counter’ (counting the number of cell divisions since the progenitor cell) to measure the number of cell divisions in a normal developmental process, that is, the production of cells in the human germline. This new approach cannot substitute classical molecular biological investigation of the germline [38] but can complement it to investigate the ancestry of a cell or tissue rather than its current state. In future, if the clock can be shown to be reliable across various tissue types, we suggest the STR clock will also be relevant for counting cell divisions that have happened in a normal process such as haemopoiesis, or in abnormal processes such as leukaemia, and cancers in general, to estimate the age of that medical condition or tumour, as advocated by Wasserstrom *et al.* [9].

The first choice for such a cancer cell replication clock in our view is the STR locus ACTBP2, because (i) unlike a SNP locus, this locus is thought to accumulate any normal mutation exclusively during a cell division; (ii) it is not prone to PCR stutter as are dinucleotide and trinucleotide STR loci; (iii) its more than 75 alleles and its complex sequence substructure allow a simple assignment of a mutant allele to a parent allele in the great majority of cases, and incidentally allows identification of operator contamination; (iv) it has a short amplicon length of about 300 nucleotides; and (v) it mutates in an order of magnitude faster (1.25% per generation) than the average tetrameric STR locus and should therefore allow the researcher or clinician to deduce the number of cell divisions between the progenitor cell and the tissue or tumour at hand from as little as a few thousand cells.

## Supplementary Material

Supplementary Table 1
